# Omouma: a prospective mother and child cohort aiming to identify early biomarkers of pregnancy complications in women living in Qatar

**DOI:** 10.1186/s12884-021-04029-4

**Published:** 2021-08-19

**Authors:** Manoj Kumar, Marwa Saadaoui, Duaa Ahmed Elhag, Selvasankar Murugesan, Shaikha Al Abduljabbar, Yassin Fagier, Osman Ortashi, Hala Abdullahi, Ibrahim Ibrahim, Medhat Alberry, Anthony Abbas, Sawssan R. Ahmed, Mohamed A. Hendaus, Karim Kalache, Annalisa Terranegra, Souhaila Al Khodor

**Affiliations:** 1 Research Department, Sidra Medicine, Doha, Qatar; 2 Obstetrics and Gynecology, Sidra Medicine, Doha, Qatar; 3 Maternal Fetal Medicine, Sidra Medicine, Doha, Qatar; 4 Psychiatry Department, Sidra Medicine, Doha, Qatar; 5 Pediatrics, Sidra Medicine, Doha, Qatar

**Keywords:** Birth cohort, Pregnancy, Multi-omics, Microbiome, Precision Medicine, Sidra Medicine, Qatar, Middle East

## Abstract

**Background:**

Pregnancy is governed by multiple molecular and cellular processes, which might influence pregnancy health and outcomes. Failure to predict and understand the cause of pregnancy complications, adverse pregnancy outcomes, infant’s morbidity and mortality, have limited effective interventions. Integrative multi-omics technologies provide an unbiased platform to explore the complex molecular interactions with an unprecedented depth. The objective of the present protocol is to build a longitudinal mother-baby cohort and use multi-omics technologies to help identify predictive biomarkers of adverse pregnancy outcomes, early life determinants and their effect on child health.

**Methods/design:**

: One thousand pregnant women with a viable pregnancy in the first trimester (6–14 weeks of gestation) will be recruited from Sidra Medicine hospital. All the study participants will be monitored every trimester, at delivery, and one-year post-partum. Serial high-frequency sampling, including blood, stool, urine, saliva, skin, and vaginal swabs (mother only) from the pregnant women and their babies, will be collected. Maternal and neonatal health, including mental health and perinatal growth, will be recorded using a combination of questionnaires, interviews, and medical records. Downstream sample processing including microbial profiling, vaginal immune response, blood transcriptomics, epigenomics, and metabolomics will be performed.

**Discussion:**

It is expected that the present study will provide valuable insights into predicting pregnancy complications and neonatal health outcomes. Those include whether specific microbial and/or epigenomics signatures, immune profiles are associated with a healthy pregnancy and/or complicated pregnancy and poor neonatal health outcome. Moreover, this non-interventional cohort will also serve as a baseline dataset to understand how familial, socioeconomic, environmental and lifestyle factors interact with genetic determinants to influence health outcomes later in life. These findings will hold promise for the diagnosis and precision-medicine interventions.

**Supplementary Information:**

The online version contains supplementary material available at 10.1186/s12884-021-04029-4.

## Background

Pregnancy is an important ‘formative period’ governed by multiple interconnected molecular processes that strongly influence the individual health trajectory from the fetal life to adulthood (Developmental Origins of Health and Disease - DOHaD) [1]. Over the course of pregnancy, the body undergoes substantial hormonal, immunological, and metabolic changes in order to promote maternal homeostasis, maintain a balanced fetal-placental interaction and support the fetal growth [2]. Although the emerging science improved our knowledge of the molecular mechanisms, and provided better therapeutic options, understanding the pathogenesis and designing a precise treatment to pregnancy complications continue to impose formidable challenges. Prominent examples are: gestational hypertension (defined as hypertension developed after 20 weeks of pregnancy) [3], gestational diabetes mellitus (GDM; defined as glucose intolerance that is first detected during pregnancy) [4], mental health disorders (defined as increased risk of depression and anxiety during pregnancy) [5], stillbirth (baby born with no signs of life at or after 28 weeks of gestation) [6], preterm birth (PTB; defined as delivery before the end of 37 weeks of gestation) [7], preeclampsia (defined as gestational hypertension, proteinuria and hyperuricemia) [8] and low birth weight (defined when the weight at birth is less than 2500 g) [9]. The emerging evidence indicates that these pregnancy complications influence the growth and development of the fetus during pregnancy and increase their susceptibility to multiple diseases later in the life [10, 11].

Recent therapeutic advances have improved pre- and post-natal care [12], but the incidence of pregnancy complications has not yet decreased. The pregnancy complications not only influence the growth of the fetus during pregnancy but also poses great health risks to the infant later in life [13]. In addition to the pre-natal period, the first 1000 days after birth represent a critical window in the newborn’s health and immune system development [14, 15]. During this window, several factors such as the mode of delivery, preterm or term birth, antibiotics usage, environmental exposure, etc., can alter or influence an individual’s immune response and propensity for developing immune disorders as well as susceptibility to chronic diseases during childhood or later [16].

Qatar has reported alarmingly high rates of non-communicable diseases (NCDs) such as diabetes, cardiovascular diseases, metabolic syndrome, hypertension and mental illness [17–20]. These percentages coincide with significantly high rates of pregnancy complications including gestational hypertension (18.4 %), GDM (21 %), pre-eclampsia (3.7 %), PTB (10 %), low birth weight (8.8 %) and still birth (6.9 %) (https://www.hamad.qa/EN/news/2019/November/Pages/HMC-Holds-Events-to-Mark-World-Prematurity-Day-2019.aspx). The etiology of these complications is highly variable and remains elusive in the majority of cases [21]. With only one birth cohort in the country [22], there is a pressing need for more research aiming to identify early biomarkers of pregnancy complications to enable the clinical team to predict and intercept complications in a precise manner.

The emerging results from the different pregnancy cohorts, such as the multi-omic microbiome study: pregnancy initiative cohort (MOMS-PI) in the American population; the longitudinal pregnancy cohort in the European population [23, 24] and the molecular signature in pregnancy (MSP) in Asian population [25, 26], along with many other studies, showed that pregnancy complications are heterogeneous, depends on multiple factors [26–32] and can also have a population-specific impact. In addition, these heterogeneous factors can also reciprocally influence each other; hence longitudinal, meta-dimensional and multi-omics approaches are required combining these heterogeneous datasets and shedding light on how different biological layers interact and contribute to the clinical outcomes. Concurrently these pregnancy cohorts were carried out within a genetically contained population; therefore, additional population-specific studies are required to capture the global picture of higher inter-individual, ethnic differences, and variable etiology of the pregnancy complications. Therefore, new studies in genetically diverse and understudied populations carrying a disproportionally higher burden of NCDs and adverse pregnancy outcomes, will help us to intercept pregnancy complications and enable personalized interventional therapies to reduce the risks of pregnancy complications.

The study name “Omouma” means motherhood in Arabic, and it implicitly explains the aim of the study. In this study, we aim to investigate factors affecting pregnancy outcomes, the early life determinants and their impact on infant, child’s and adolescent’s health in a geographically and genetically diverse population. We will monitor the pregnant women every trimester until delivery and one-year post-partum. Using the biological samples collected from the mother-baby pairs, we aim to identify the molecular mechanisms of pregnancy complications, such as GDM, gestational hypertension, preeclampsia, stillbirth and preterm birth, and to identify biomarkers that can help predicting pregnancy outcomes and may pave the way for a personalized intervention. We will also investigate the maternal, genetic, social, environmental, lifestyle on the infant’s health and how early childhood determinants affects health outcomes of the infant, child and adolescent.

## Methods/design

### Omouma cohort description

**Fig. 1:**
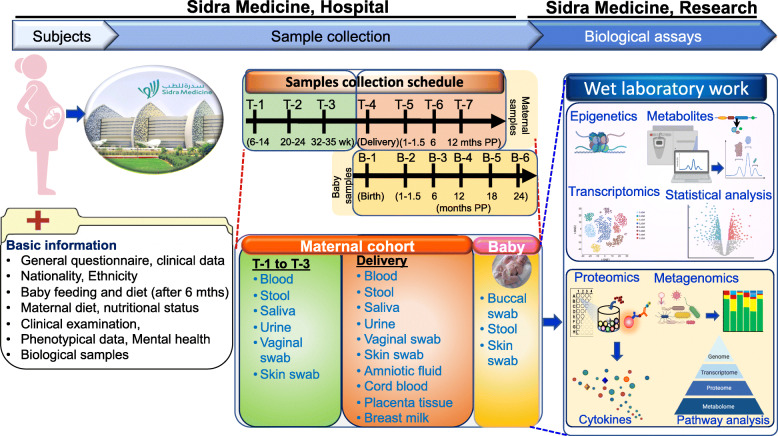
Study overview and timeline of sample collections. Mths: Months; T: Timepoints for maternal sampling; B: Timepoints for baby sampling; PP: postpartum

Social, environmental, nutritional, genetic factors and human-associated microbial communities play an important role in human health and disease and may contribute to the pregnancy outcomes and even neonatal health and well-being. To explore this hypothesis, a prospective Omouma cohort study was designed at Sidra Medicine, Qatar with the aim to recruit 1000 pregnant women and their neonates to comprehensively investigate maternal and infant factors in relation to child health outcomes, as well as early-life determinants of NCDs (Fig. 1).

All the pregnant women attending Sidra Medicine for their antenatal care management (ANC), in their first trimester, and planning to deliver at Sidra Medicine will be reviewed by qualified physicians. Potential eligible women will be approached and informed about the background of the study by a trained coordinator. The gestational age will be determined using early ultrasound scan by trained technicians. Women in the first trimester (6-<14 weeks of gestation) will be included in this study. The inclusion and exclusion criteria are summarized below.

#### Inclusion criteria

- Pregnant woman able and willing to provide informed consent for herself as well as approving her neonate to be included in the study.
- In her first trimester (6-<14 weeks) with singleton pregnancy.
- Any nationality or ethnic group.
- Age ≥ 18 years.
- Planning to deliver at Sidra Medicine.
- Willing to provide all study samples and comply with the study requirements.

#### Exclusion criteria

- Age < 18 years.
- Non-pregnant.
- Non-viable pregnancy at the time of recruitment.

The primary goal of the Omouma cohort is to identify predictive biomarkers associated with adverse pregnancy outcomes by designing multiple molecular assessments of the samples collected during pregnancies. Those molecular profiles will be compared between healthy and complicated pregnancies in order to identify early predictive biomarkers for pregnancy complications. We will also extensively follow up the baby for the first two years of life, to assess how health during pregnancy will influence children’s health and development. At present, recruitment of study participants is on-going.

### Ethics approval, consent to participate and withdrawal

The study protocol and various questionnaires designed to collect demographical, clinical and nutritional information are approved by the Institutional Review Board (IRB) of Sidra Medicine under (IRB protocol #1603700). Written informed consent is obtained from all participants prior to their enrollment in the study. All experiments are performed in accordance with the approved guidelines. Study participants are informed about their fundamental rights, including the study withdrawal. Participation in this study is voluntary, therefore any participant can withdraw from the study for any reason without penalty or loss of benefits to which they are otherwise entitled and without any effect on their future medical care.

### Sample size calculation

The sample size calculation and feasibility of study were performed considering the emerging incidence of NCDs during the reproductive age of women in the state of Qatar and the disproportionally higher burden of adverse pregnancy outcomes. Assuming a GDM rate of 20 %, PTB rate of 8.5 % and a sample sensitivity of 80 %, the calculated sample size needed for this study is proposed to be 1000 subjects. All power calculations of the main effects assume a two-sided alpha of 0.05 and a power of 80 %. Calculations were done using the CDC EpiInfo program version 7.0 and applying the Fleiss test [33].

### Sampling procedures

The samples will be collected from the study participants during each trimester of pregnancy, at the time of delivery and post-delivery. In addition, multiple samples will be collected from the newborns. An overview of the study protocol and details of various sampling are summarized in Fig. 1. Tables 1 and 2 describe the sample timing and collection procedure.

**Table 1:** Study timeline and sample collection details from women during pregnancy, at delivery and post-partum and sample collection from neonates

Sample type/ Time points	Samples collection from women								Samples collection from neonate					
	T1	T2	T3	Del-T4	PP- T5	PP- T6	PP- T7	UE	B1	B2	B3	B4	B5	B6
Blood	+	+	+	+	+	+	+	+	+	+	+	+	+	+
Stool	+	+	+	+	+	+	+	+	+	+	+	+	+	+
Saliva^a^	+	+	+	+	+	+	+	+	+	+	+	+	+	+
Urine	+	+	+	+	+	+	+	+	+	+	+	+	+	+
Vaginal Swab	+	+	+	+	+	+	+	+	-	-	-	-	-	-
Skin swab	+	+	+	+	+	+	+	+	+	+	+	+	+	+
Amniotic fluid	-	-	-	+	-	-	-	-	-	-	-	-	-	-
Cord blood	-	-	-	+	-	-	-	-	-	-	-	-	-	-
Placenta	-	-	-	+	-	-	-	-	-	-	-	-	-	-
Breast milk	-	-	-	-	+	+	+	-	-	-	-	-	-	-

^a^for neonate, a buccal swab will be collected

T1: Trimester 1 (6–14 weeks); T2: Trimester 2 (20–24 weeks); T3: Trimester 3 (32–35 weeks); Del-T4: Delivery; PP-T5: post-partum (4–6 weeks post-partum); PP-T6: post-partum (6 months post-partum); PP-T7: post-partum (12 months post-partum); UE: Unwell Episode; B1: Birth (day 0); B2: Birth (1-1.5 months); B3: Birth (6 months); B4: Birth (12 months); B5: Birth (18 months); B6: Birth (24 months); +: sample collection; -: no sample collection

**Table 2:** various sample types and their collection and storage procedure from women and their neonates

Sample type	Collection procedure	Applications
Blood sample	A small blood sample will be collected into three tubes during each antepartum visit. (a). Three ml blood will be collected in tempus tube and will be used for epigenetic studies. (b). Four ml blood will be collected in EDTA tubes and from this 1 ml blood will be mixed with 10 % DMSO and stored at -80 °C for cell sorting; another 1 ml blood will be used for plasma separation and stored at -80 °C; while remaining, 2 ml will be used for stimulation with Toll-like receptors ligands and after stimulation, the plasma samples will be stored at -80 °C. (c). Three ml blood sample collected in EDTA tube will be used for Peripheral Blood Mononuclear Cells (PBMC) separation and isolated PBMC will be preserved in preservative medium and stored in liquid nitrogen.	Blood transcriptomics, DNA genotyping, Immune stimulation, Cell sorting, PBMC isolation, plasma isolation
Vaginal swab	A vaginal swab sample will be collected from the posterior fornix using a soft Copan eswab and stored at -80 °C.	Metagenomics
Skin swab	A skin swab from the upper chest area will be collected during each visit using a BD eswab collection and transport kit and stored at -80 °C.	Metagenomics
Saliva/ buccal swab	A saliva sample will be collected in a sterile 50 ml falcon tube. The sample will be aliquoted and stored at -80 °C.	Metagenomics
Urine sample	A urine sample will be collected in a sterile 50 ml container. The sample will be aliquoted and stored at -80 °C.	Proteomics, Metagenomics, Metabolomics
Stool sample	A fresh stool sample will be collected and split into two tubes (a). DNAGenotek Omnigut tube and (b) cryotube and will be stored at -80 °C.	Metagenomics, Metabolomics
Amniotic fluid	An amniotic fluid sample will be collected at delivery and stored at -80 °C.	Proteomics, Metagenomics, Metabolomics
Cord blood	A cord blood sample will be collected at delivery and stored at -80 °C.	Epigenetics, miRNA purification
Placenta	A placenta sample will be collected at delivery and stored at -80 °C.	Metagenomics, Epigenetics
Breast milk	A small breast milk sample around 1 ml will be collected during post-partum visits as described in Table 1.	Metagenomics

Blood samples will be collected at each visit by a trained phlebotomist. Saliva, urine, skin swab from the upper chest area (2 × 2 cm) and stool sample will be self-collected during each visit, at delivery and at the follow-up post-partum visits. Vaginal samples will be self- collected or collected by a clinician [34]. Amniotic fluid will be collected during pregnancy only if clinically indicated. Cord blood and placenta tissue will be collected in stile conditions at the time of delivery. Pregnant women who will have an unwell episode during pregnancy, will have routine tests for clinical investigation and a set of biological samples will be collected for the study (Table 1).

**Fig. 2:**
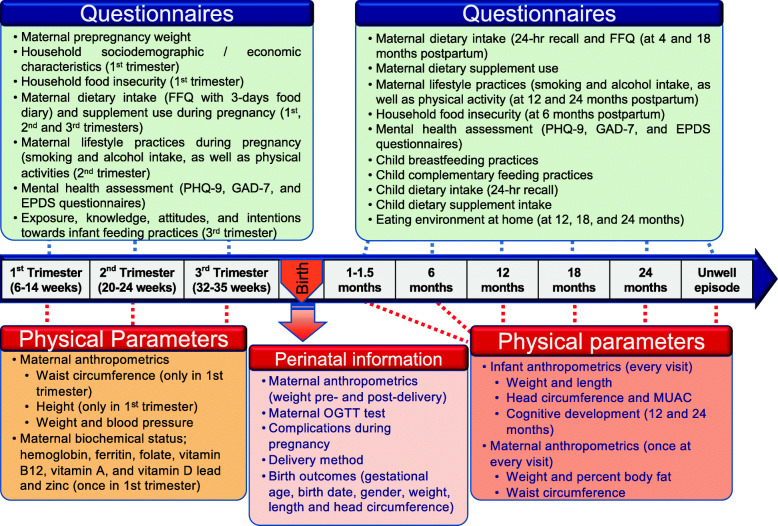
Demographic and physical data collection details during the course of the study. FFQ: Food Frequency Questionnaires; OGTT: oral glucose tolerance test; MUAC: Mid-Upper Arm Circumference; PHQ-9: Patient Health Questionnaire-9; GAD-7: Generalized Anxiety Disorder-7; EPDS: Edinburgh Postnatal Depression Scale

Blood, stool, urine, buccal and skin (chest area) swabs will be collected from all the neonates from term and preterm deliveries at birth and during their follow-up visits as described in Table 1. Over 75,000 biological samples will be collected during this study and deposited into the Sidra research repository for future research. Besides, details of the pregnancy including obstetric and medical complications will be recorded (Fig. 2).

### Maternal socio-economical and nutritional assessment

Socio-economical assessment of the enrolled pregnant women will be performed at the time of recruitment using a socio-economic questionnaire (supplementary file 1). Any history of previous illness, allergies, chronic diseases will be recorded in the first visit. Weight, height, blood pressure, urine analysis, and any pregnancy complications data will be recorded at each visit. In addition, use of medications, including antibiotics and supplements will be recorded.

Dietary intake will be assessed during their clinical visits as described in Fig. 2, using different questionnaires: 24 h dietary recall (supplementary file 2) and 3-days food diary (supplementary file 3). Food consumption will be self-reported by recording 3 days diet intake (2 routine weekdays and one weekend/festive day per week) on a diary provided by the research coordinator. The 24 h recall collected by trained staff will serve to validate the 3 days diary self-reported by the subject. Dietary information will be used as contributing factor to the multi-omics signature modelling approach. Food nutrients composition will be calculated by analyzing the 3 days diaries and the 24 h recalls with Nutritionist Pro software (Axxya Systems, https://www.nutritionistpro.com/).

### Mental health assessment

Mental health status, including anxiety and depression symptoms, will be assessed using two questionnaires: the Patient Health Questionnaire-9 (PHQ-9) and the Generalized Anxiety Disorder-7 (GAD-7) scales during pregnancy, after delivery and 6 or 12 months postpartum (Fig. 2) [35, 36]. Depression symptoms will be assessed during pregnancy and postpartum using the Edinburgh Postnatal Depression Scale (EPDS) questionnaire [37]. Women who endorse the suicidality question will be offered a consultation by a clinician at Sidra Women’s Mental Health Clinic who will ascertain the level of risk and helps accordingly.

### Infant feeding and dietary intake

Breastfeeding and complementary feeding practices of infants will be assessed using a questionnaire and will be categorized as exclusive breastfeeding, predominant breastfeeding, or complementary feeding. To evaluate infants’ complementary feeding, mothers will be asked about the timing of introduction and type of complimentary food, i.e., solid, semi-solid, and soft foods, including minimum meal frequency, adequate diet, and consumption of iron-fortified or iron-rich foods.

### Infant cognitive development

The infants’ cognitive development will be assessed using different childhood follow-up questionnaires described in Fig. 1 [38]. The questionnaire will be completed based on parents’ assessment at 12- and 24-months ages, but if required, it may also be completed by a professional interacting with the parent and the infant. The questionnaire is highly specific, sensitive, reliable, and is widely used in pediatric populations as a developmental screening tool [39]. These parameters will be assessed by the pediatrician as described earlier [38].

### Infant anthropometric assessment

The infants’ anthropometric assessment will be performed during their follow-up visit as described in Fig. 2, following standard techniques by nurses at Sidra hospital. The infant’s anthropometric measurements will include head circumference, length, weight, and mid-upper arm circumference (MUAC). Following the child growth standards of WHO [40], the infants will be categorized into stunting, wasting, underweight, overweight, and obesity [41]. Besides, MUAC measurement will be used to assess the nutritional status of infants.

### Multi-Omics assays

The healthcare challenges imposed by complicated pregnancy cannot be resolved by traditional therapeutic, clinical, and investigational approaches, as these approaches can only examine few facts of a much complex problem. To address these challenges, we will longitudinally characterize the phenotype of complicated and uncomplicated pregnancy by integrating multilevel multi-omics profiles using advanced high throughput technologies to capture a wide range of molecular and cellular processes along with environmental parameters of all individuals. The first tier of multi-omics data in this study (Fig. 1; Table 1) will include longitudinal profiling of blood-immune transcriptomics, proteomics, metabolomics, epigenomics, and metagenomics. Improving our understanding of complicated pregnancy will finally require analysis of these datasets independently and then integrating the various datasets together to reveal the pathogenetic networks and identify predictive biomarkers.

#### Immune stimulation and blood transcriptomics

Whole blood (WB) samples will be collected at different time-points during pregnancy, on delivery and during postpartum (as shown in Table 1). The blood samples will be aseptically processed for different applications as described in Table 2 and stored in a -80 °C freezer until further analysis. The blood cells will be lysed, and total RNA was extracted using the Paxgene Blood RNA kit (Qiagen, MD) according to the manufacturers protocol. RNA integrity and concentration will be assessed with a Bioanalyzer (Agilent Technologies), and transcriptional responses will be measured using a high throughput PCR platform (Fluidigm BioMark HD) as previously described [42].

#### Proteomics and metabolomics

Blood and vaginal cytokines profiles during pregnancy, will be evaluated using Bio-Rad Bio-Plex Pro human cytokine Multi-Plex assay kit (Bio-Rad Laboratories, Inc., USA) with a Luminex 3D system. Selected stool samples will be used for targeted and untargeted metabolomic profiles using advance analytical chemistry technique that combines the physical separation capabilities of liquid chromatography (or HPLC) with the mass analysis capabilities of mass spectrometry (MS).

#### Epigenomics

Blood, placenta, and maternal cord blood collected will be subjected for epigenomics measurements using Illumina Epic DNA methylation array. Briefly, DNA will be extracted from whole blood samples using the QIAamp DNA micro kit (Qiagen, MD) as described earlier [43]. DNA (250–750 ng) will be treated with sodium bisulphite using the EZ DNA methylation kit (Zymo Research, CA, USA) and DNA methylation will be performed using Illumina Epic DNA methylation kit (Illumina, CA, USA) and processed on Illumina IScan using the manufacturer’s standard protocol. All downstream analysis will be conducted using the hg19/GRCh37 human genome assembly as described earlier [43].

#### Metagenomics

Microbial genomic DNA will be extracted from different biological samples (Table 2) using QIAamp DNA mini kit (Qiagen, MD) as previously described [44]. The extracted DNA samples will be stored at -20 °C until further processing. The quantity of DNA samples will be assessed using NanoDrop spectrophotometer (Thermo Scientific). All samples will be processed for metagenomics analysis using an Illumina MiSeq 2 × 300 platform (Illumina, Inc. San Diego) at Sidra as previous described [26, 45]. For the microbiome analysis, the demultiplexed FASTQ files will be converted into FASTA files and analyzed using QIIME 2 software. Operational taxonomy units (OTUs) will be generated by aligning against SILVA 16 S rRNA database as a reference [46]. Alpha diversity will be calculated using Phyloseq package in R platform [47]. Beta diversity will be calculated using Unifrac distance method for all microbial communities of the sample through principal coordinates analysis [48].

### Statistical and integrated analysis

Emerging evidence already indicates that the challenges imposed by the complex diseases can be met using the advance multi-omics analysis of different variable factors and applying integrative bioinformatic tools for identifying the interacting molecular networks [28]. Hence, in the present cohort, the burden of adverse pregnancy outcomes in relation to nutritional exposures, environmental, lifestyle will be analyzed by integrating the various omics datasets, generated from this cohort. An appropriate statistical tool such as R and SAS v9.4 (SAS Institute) will be applied to analyze the individual omics datasets to capture trends as well as variations in each dataset. Statistical analyses will include both descriptive (numerical and graphical) and inferential statistics.

Graphical analyses, where deemed appropriate, will be included in the analyses. The statistical analyses are directed towards the assessment of the objectives of the study. Given the large number of markers that are anticipated to be identified, principal components analysis (PCA) technique will be used first to identify patterns and trends of various factors within and between the cases and controls.

**Fig. 3:**
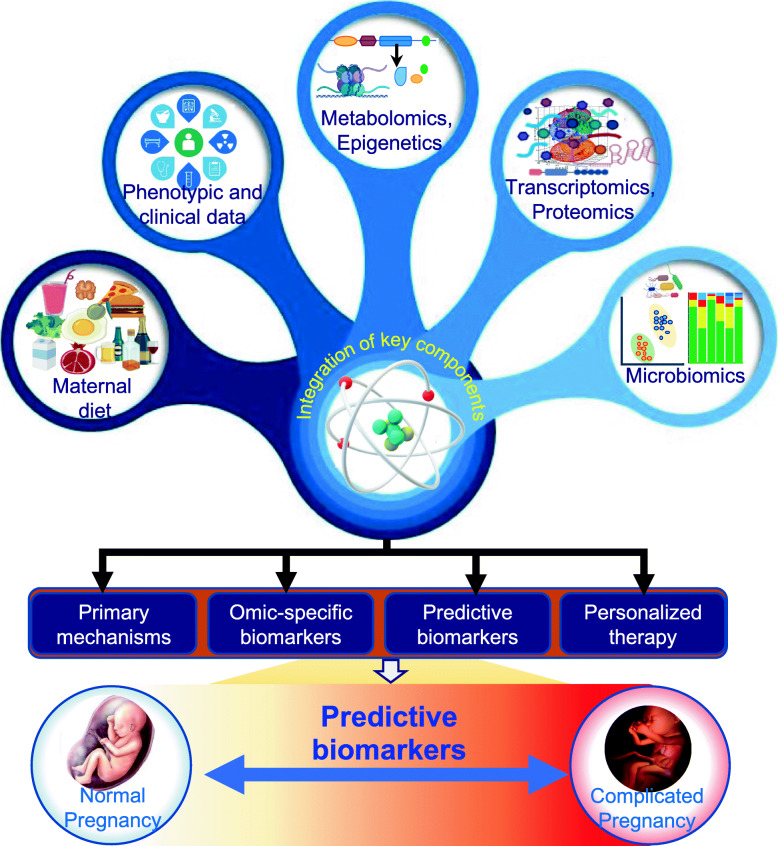
A multi-omics approach to predict pregnancy outcomes. The datasets from various omics will be used and integrated for biomarkers discovery

A correlation analysis will be applied to integrate different omics datasets as well as clinical and environmental parameters to understand the strength and depict directionality of relationships between different datasets. Finally, statistically significant co-variable identified from single omics datasets will be integrated together to build predictive biomarkers for monitoring the pregnancy complication (Fig. 3). For the predictive model, we will use supervised methods namely: partial least squares discriminate analysis (PLS-DA), logistic regression, support vector machines (SVMs) and the Random Forest [49].

## Discussion

This study outcomes may have major implications on the clinical management of pregnant women in the state of Qatar. The main goal of this longitudinal cohort is to apply a multi-omics approach to understand the multifactorial causes of pregnancy complications and avoid the pitfalls generated by other studies that evaluated these factors separately [50–52]. The project will serve primarily to identify potential predictive diagnostic biomarkers for the early detection of pregnancy complications, as well as infant health and early-life causes of NCDs. The merits of evaluating the multi-omics approach are attributed to the current challenges intrinsic to intercept pregnancy complications.

### Environmental factors

Indirect but substantial evidence indicates that multiple complex stimuli represented either by the external environment (exposome; diet, lifestyle, stress level etc.) or by the internal microbial environment (vaginal and gut microbiome), can influence each other, as well as affect the molecular mechanisms in a reciprocal fashion [53, 54]. Unfortunately, modern lifestyle and changing in the dietary habits are associated with the increasing prevalence of NCDs in Qatar [17], and affecting other environmentally induced factors, such as host-epigenetic or microbiome, might contribute to pregnancy complications or adverse pregnancy outcomes [51, 53, 55]. Given that a single environmental stimulus may have a substantial effect on pregnancy, such as diet, stress, environmental factors, and microbiome composition, understanding the complexity of these interactions remains a challenge [54, 56, 57].

### Blood transcriptomics, proteomics and metabolomics factors

In the recent years several studies have shown the importance of blood transcriptomics during pregnancy to predict pregnancy complications [42, 58]. However, the interpretation of gene transcription data must be cautious, because of the considerable variability between individuals, populations, ethnic groups and the poor correlations between mRNAs and protein levels [25, 42], hinders the identification of common predictors. In addition, recent studies showed that the vaginal microbiome might influence the vaginal environment during the course of pregnancy by secreting proteins that interact or bind with host proteins to modulate different biological functions and influence pregnancy outcomes [59]. Similarly, metabolic profiling studies reported differences between women with complicated pregnancy and healthy pregnancy. In the current cohort, a blood transcriptomics, proteomics and metabolomics signatures during the course of pregnancy and at birth will be evaluated to reveal molecular trajectories precede to pregnancy complications.

### Epigenomics factors

Technological advances in the last two decades have enabled the assessment of multiple constitutive elements simultaneously and enable us to identify not only the disease-associated gene but also revealed the vital importance of non-genetic variants in the regulation of gene expression. This non-genetic modification, also known as epigenetic modification, can alter the structure of chromatin from active to repressed by methylation or histone modification and can influence how, when and where genes are expressed [60, 61]. By profiling the non-genetic modifications, we can provide a whole new insight into the molecular basis of pregnancy complications. Altered epigenetic profiles have been observed in suboptimal maternal conditions, such as GDM, obesity, or pre-eclampsia [50, 62]. Hence, the epigenetic comparisons during a healthy pregnancy and complicated pregnancy can lead us to identify specific signatures well before the clinical manifestations. Moreover, we will investigate if any specific signature will be transmitted from the mother to the baby.

### Microbiomics

Growing evidence suggests the influencing role of microbiome to the health of pregnant women and their neonates during pregnancy and beyond [15, 28]. Even post-delivery, babies get major microbial inoculum through the breast milk, which can stimulate the intestinal immune homeostasis, including microbial communities of neonates to fight against immune-mediated diseases later in life (such as asthma, allergies, type 1 diabetes etc.) [63]. The microbiome consists of discrete microbial communities dominated by different bacterial taxa. In addition, the dynamics of microbiome composition undergoes considerable changes during pregnancy and also exhibits clade-specific depletion or enrichments that could vary depending on ethnicity [28, 29]. For example, a vaginal microbial community dominated with *Lactobacillus* species has been associated with a healthy pregnancy, whereas the abundance of *Gardnerella*, *Prevotella* and bacterial vaginosis (BV)-associated bacterium-I (BVAB-I) has been associated with adverse pregnancy outcomes in Caucasian women [64]. In addition, gut microbial communities also undergo substantial changes to support nutrient acquisition and fetal growth during pregnancy [65, 66]. However, a definitive conclusion from these studies is still missing as the pathophysiology of pregnancy complications is heterogeneous, and gut and vaginal microbial communities are highly variable. Moreover, the ethnic difference in the microbial communities hinders the identifications of specific microbial communities associated with a specific pregnancy complication [26, 67]. Therefore, it is vital to identify microbial dynamics associated with specific pregnancy complications in the Qatar population, which is an unexplored ethnic group, and integrate the microbiome composition with other -omics datasets to reveal the role of microbiome in pregnancy outcomes.

### Solving the complexity of pregnancy complications

Multifactorial processes, including pregnancy complications are the results of multiple biological actions mediated by dynamic, cellular, immunological, and molecular networks together with complex, erratic and nonlinear responses that depend on the number of cross-talks events within the networks.

Pregnancy complications cannot be solved by the conventional approaches. There is a need to develop alternative approaches to enhance our understanding of their pathophysiology, intervention, interception, and precise treatment. State-of-the-art multi-omics approaches are needed to improve our understanding of the complex interaction governing pregnancy complications. In addition, the development of novel bioinformatics tools has revolutionized the biomarker discovery field. The first step in the multi-omics approach is the identification of the key interacting components from each omics, the second step is the functional characterization of each key element, the third step is the integration of the all-omics components to understand the structure or role of each component in pathogenesis and the final step will be the evaluation of the response of various key drivers or biomarkers. Successful completion of the Omouma cohort will yield an unprecedented wealth of data related to pregnancy complications and will pave the way towards new diagnostic and therapeutic opportunities used to better monitor and treat pregnancy complications. In addition, in the Omouma study, we will learn how the maternal health during pregnancy affects neonatal and child health outcomes.

### Strengths and limitations of the study

The main strength of this study protocol is inherited by its prospective design, which is optimized to evaluate the performance of multi-variable factors aiming to capture the dynamic changes during pregnancy and identify predictive biomarkers of pregnancy complications. Another major strength is the high frequency sampling and follow-up of pregnant women who actually carry a high risk of pregnancy complications. The unique high-frequency sampling during and post-pregnancy will allow the evaluation of rare exposures and track the transition, pattern, and change in exposure over time during pregnancy and post-partum. The multi-disciplinary datasets generated from this cohort will facilitate the clinical management of high-risk pregnancies in high-resource settings, where early identification of pregnancy complications has a real chance of improving the outcome for mother and child. Previous mother and child cohorts have focused more on Western or Caucasian populations, but this ethnically diverse, mostly Arab population has so far been “lost in the noise”.

In contrast to these major strengths, this study protocol may have some potential limitations, such as lost follow-up or protocol deviations. Due to long-term follow-up and high frequenting sampling, the loss of some pre-protocol samples or even study participants are expected. So, we will replace the study subjects who are lost before delivery with new study participants. The sample size was calculated by considering 20 % attrition rate. Multi-ethnic population can introduce high variance in the data results. We will analyze the genetic background and the cultural differences collecting socio-economic, nutritional and lifestyle information.

## Supplementary Information

**Additional file 1.**

**Additional file 2.**

**Additional file 3.**

## Data Availability

Not applicable.

## Abbreviations

CDC: Centers for Disease Control and Prevention
GDM: Gestational Diabetes Mellitus
DOHaD: Developmental Origins of Health and Disease
PTB: Preterm Birth
IRB: Institutional Review Board
MUAC: Mid-Upper Arm Circumference
WHO: World health Organization
WB: Whole Blood
RNA: Ribonucleic Acid
DNA: Deoxyribonucleic Acid
MOMS-PI: Multi-Omic Microbiome Study: Pregnancy Initiative cohort
MSP: Molecular Signature in Pregnancy
ANC: Antenatal Care Management
PHQ-9: Patient Health Questionnaire-9
GAD-7: Generalized Anxiety Disorder-7
EPDS: Edinburgh Postnatal Depression Scale
TLR: Toll Like Receptors
OTUs: Operational taxonomy units
QIIME: Quantitative Insights Into Microbial Ecology
PCA: Principal Components Analysis
BVAB-I: Bacterial Vaginosis (BV)-Associated Bacterium-I
HPLC: High Performance Liquid Chromatography
MS: Mass Spectrometry
PLS-DA: Partial Least Squares Discriminate Analysis
SVMs: Support Vector Machines
NCDs: Non-Communicable Diseases
