# Supplementary material for: Omouma: a prospective mother and child cohort aiming to identify early biomarkers of pregnancy complications in women living in Qatar
Source: BMC Pregnancy Childbirth. 2021 Aug 19;21:570. doi: 10.1186/s12884-021-04029-4 (PMC8377974; doi:10.1186/s12884-021-04029-4)
Supplement: Supplementary file 1 — Additional file 1. [file 12884_2021_4029_MOESM1_ESM.pdf]

Study title: امومة" Omouma": The Mother and Child Cohort at Sidra Medicine

1. **Age**: -----------------------------------

2. **Nationality:** ---------------------------------

3. **Ethnicity:**
- ❑ Qatari-Bedouin descendant
- ❑ Qatari-Persian descendant
- ❑ Qatari-African descendant
- ❑ Qatari-unknown
- ❑ White
- ❑ African
- ❑ Asian
- ❑ Arab
- ❑ More than one race:------------
- ❑ Refused / Unknown

4. **Marital Status**
   - ❑ Married
   - ❑ Divorced

5. **Number of family members living with you** (children, spouse, parents): -------------------------------

6. **Age of your first pregnancy**: ---------------------------------

7. **Number of previous pregnancies**: ---------------------------------

8. **Number of kids you have**
   - ❑ 0
   - ❑ 1-2
   - ❑ ≥3.   How many: --------------------

9. **Education level**
   - ❑ Secondary school degree
   - ❑ Bachelor's degree
   - ❑ Master's degree
   - ❑ PhD or higher
   - ❑ Other

10. **Employment**
    - ❑ Not working
    - ❑ Working, specify type of job: ---------------------------------

11. **Pets in the house**
    - ❑ No
    - ❑ Yes, which type of animal? ---------------------------------

12. **Smoking:**
    - ❑ No
    - ❑ Yes, how many times per day? And what do you smoke? __________
    - ❑ Do you consider yourself a secondary smoker? If anyone living with you
