# Supplementary material for: Omouma: a prospective mother and child cohort aiming to identify early biomarkers of pregnancy complications in women living in Qatar
Source: BMC Pregnancy Childbirth. 2021 Aug 19;21:570. doi: 10.1186/s12884-021-04029-4 (PMC8377974; doi:10.1186/s12884-021-04029-4)
Supplement: Supplementary file 2 — Additional file 2. [file 12884_2021_4029_MOESM2_ESM.pdf]

24HDR

**Patient ID**:	
**Dates of recorded intake:**	
**Nutritionist:**	
**Nutritionist Contact Information:**	

Instructions for Keeping Your 24Hrs Food Record

- Please keep your 24hr food record for one day.
- Select day that closely resemble your usual eating habits.
- Each time you eat or drink anything (meals, snacks, etc.) during the day, write down what and how much was served and what and how much was eaten.
- To measure how much was eaten, use a set of **measuring cups and spoons** to help estimate amounts. Also see the examples below to estimate portion sizes.
- Note if food choices are homemade or purchased. Please include brand names whenever possible.

Amounts and Conversions

1/4 cup = 50 ml or 4 Tablespoons

1/3 cup = 75 ml or 5 1/2 Tablespoons

1/2 cup = 125 ml or 8 Tablespoons

2/3 cup = 150 ml or 10 1/2 Tablespoons

3/4 cup = 175 ml or 12 Tablespoons

1 cup = 250 ml or 16 Tablespoons

1 glass = 200 ml

30 gr = 1 slice of processed cheese or lunchmeat

How to Estimate Your Portion Size

**Meat**<br>Ninety (90) gr of meat are about the size and thickness of a deck of playing cards or an audiotape cassette	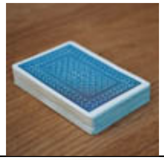
**Fruit**<br>A medium apple or peach is about the size of a tennis ball	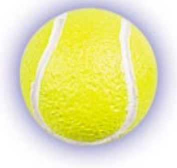
**Grains**<br>One cup of rice or pasta is about the size of your fist	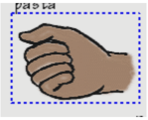
**Cheese**<br>Thirty (30) gr of cheese is about the size of four dice	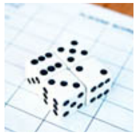

Three-Day Food Record Checklist

**Beverages**	What kind of milk? Homo, 2%, 1%, skim, other.<br>Was it fruit juice or fruit beverage or drink?
**Breads**	Did you spread on butter or margarine?
**Cereal**	Did you add milk?<br>Did you add sugar or fruit?
**Dairy**	What brand or kind of yogurt?<br>What brand or kind of cheese?
**Vegetables**	Was it raw or cooked?<br>Was it fresh, frozen or canned?<br>Did you add any butter, margarine or sauce?
**Fruit**	Was it a small, medium or large fruit?<br>Was it fresh, frozen or canned?
**Grains**	Did you add any butter, margarine, peanut butter, jam or honey?<br>Was it a half or whole sandwich?<br>Was it a small or large muffin or bagel?
**Fish**	Was your canned fish packed in water or oil<br>How did you cook your fish?
**Meats**	How did you cook your meat?<br>What kind of cut was it e.g. chicken leg or chicken breast?
**Soups**	Was your soup prepared with milk, water or cream?
**Restaurants**	What restaurant was it?
**Packaged food**	What brand was it?

SAMPLE MENU – 24 HRS

Day 1: Tuesday, May 14, 2015				
**Time of Meal or Snack**	**Type of Food or Beverage Offered**	**Amount Eaten**	**Method of Preparation or Brand**	**Comments** (e.g. amount of food served, too tired to eat)
**Breakfast**	Cereal	½ cup	Honey Nut Cheerios	
	Milk 2%	½ cup		On cereal
	Banana	½ med		
**AM Snack**	Animal Crackers	10	Christie	
	Apple juice	120 gr	Allen's pure apple juice-canned	
**Lunch**	Grilled cheese sandwich			
	Whole wheat bread	1 slice	Dempsters	No crusts
	Cheese slice	1 slice	Kraft slices	
	Butter on bread	1 Tbsp		
	Yogurt – strawberry	75 ml	Mini-go	
	Milk	½ cup	2%	
**PM Snack**	Granola bar	1 bar – 35 g	Quaker Chewy, Trail Mix – tropical fruit	Ate half of it
**Dinner**	Chicken fingers	1 ½	President's Choice	
	French fries	10	McCain regular	
	Honey	2 Tbsp		For dipping
	Ketchup	2 Tbsp	Heinz	
	Carrots	½ medium	Raw, cut in sticks	
	Milk	½ cup	2%	
**Evening Snack**	Ice cream	1 cup	Chocolate Nestle	

Was this day's intake considered: [ ] Poor [X] Average [ ] Very Good

24 HRS

Date:

Time of Meal or Snack	Type of Food or Beverage Offered	Amount Eaten	Method of Preparation or Brand	Comments (e.g. amount of food served, too tired to eat)
**Breakfast**				
**AM Snack**				
**Lunch**				
**PM Snack**				
**Dinner**				
**Evening Snack**				

Was this day's intake considered: [ ] Poor [ ] Average [ ] Very Good
